# Environment Symmetry Drives a Multidirectional Code in Rat Retrosplenial Cortex

**DOI:** 10.1523/JNEUROSCI.0619-22.2022

**Published:** 2022-12-07

**Authors:** Ningyu Zhang, Roddy M. Grieves, Kate J. Jeffery

**Affiliations:** Institute of Behavioural Neuroscience, Division of Psychology and Language Sciences, University College London, London WC1H 0AP, United Kingdom

**Keywords:** complex environments, head direction cells, hippocampal system, retrosplenial cortex, spatial cognition, symmetry

## Abstract

We investigated how environment symmetry shapes the neural processing of direction by recording directionally tuned retrosplenial neurons in male Lister hooded rats exploring multicompartment environments that had different levels of global rotational symmetry. Our hypothesis built on prior observations of twofold symmetry in the directional tuning curves of rats in a globally twofold-symmetric environment. To test whether environment symmetry was the relevant factor shaping the directional responses, here we deployed the same apparatus (two connected rectangular boxes) plus one with fourfold symmetry (a 2 × 2 array of connected square boxes) and one with onefold symmetry (a circular open-field arena). Consistent with our hypothesis we found many neurons with tuning curve symmetries that mirrored these environment symmetries, having twofold, fourfold, or onefold symmetric tuning, respectively. Some cells expressed this pattern only globally (across the whole environment), maintaining singular tuning curves in each subcompartment. However, others also expressed it locally within each subcompartment. Because multidirectionality has not been reported in naive rats in single environmental compartments, this suggests an experience-dependent effect of global environment symmetry on local firing symmetry. An intermingled population of directional neurons were classic head direction cells with globally referenced directional tuning. These cells were electrophysiologically distinct, with narrower tuning curves and a burstier firing pattern. Thus, retrosplenial directional neurons can simultaneously encode overall head direction and local head direction (relative to compartment layout). Furthermore, they can learn about global environment symmetry and express this locally. This may be important for the encoding of environment structure beyond immediate perceptual reach.

**SIGNIFICANCE STATEMENT** We investigated how environment symmetry shapes the neural code for space by recording directionally tuned neurons from the retrosplenial cortex of rats exploring single- or multicompartment environments having onefold, twofold, or fourfold rotational symmetry. We found that many cells expressed a symmetry in their head direction tuning curves that matched the corresponding global environment symmetry, indicating plasticity of their directional tuning. They were also electrophysiologically distinct from canonical head directional cells. Notably, following exploration of the global space, many multidirectionally tuned neurons encoded global environment symmetry, even in local subcompartments. Our results suggest that multidirectional head direction codes contribute to the cognitive mapping of the complex structure of multicompartmented spaces.

## Introduction

Self-localization and navigation require construction of a stable directional signal that orients the map of space in the brain to be in register with the actual environment. Retrosplenial cortex (RSC) is a brain region where internal and external orientation signals combine, as evidenced by the long-established existence of head direction cells (Cho and Sharp, 2001), which are compass-like, directionally tuned neurons that fire according to the facing direction of the animal. Recent findings revealed that in addition to these classic head direction (HD) cells (Taube, 1995) in dysgranular RSC (dRSC), there are also landmark-sensitive bidirectional (BD) cells that were found to express a bipolar firing pattern if recorded in a two-compartment space having twofold rotational symmetry (Jacob et al., 2017). This bidirectional pattern was surprising, having not been noted previously. We hypothesized that it arose from experience in the unusual environment structure of our experiment and suggested that it reflects a general propensity for the cells to detect, and acquire encoding of, environment symmetry. To test this, in the present experiment we recorded RSC neurons in environments having symmetries that were onefold, twofold, and fourfold. We found that a subpopulation of electrophysiologically and anatomically distinct directionally tuned neurons showed multidirectional tuning that accorded with the symmetry of the environment, not just globally, when considered across the whole environment but also sometimes locally within each subcompartment. This pattern persisted in the dark and was not due to occult egocentric boundary encoding. Global multidirectional encoding indicates tuning of the neurons to the local environment polarities (and hence is only multifold when all the compartments are considered together), whereas local encoding of global symmetry means that the global multifold symmetry experienced after exploration has been captured by the network and is re-expressed locally. This enables spatial encoding of the environment beyond immediate perceptual reach, which may be important in building a cognitive map of a multicompartment space.

## Materials and Methods

### Subjects

A total of 18 adult male Lister hooded rats (Charles River Laboratories) were used in experiments in the study, 14 in the 2-box environment, 8 in the 4-box environment, with 4 that had experience in both types of boxes. All 18 animals also explored the 1-box environments. They were individually housed in a temperature- (22 ± 2°C) and humidity-controlled (55 ± 10%) holding room with a 12 h light/dark cycle, with 1 h of simulated dusk/dawn. A hanging hammock or nesting box was provided as enrichment. Animals received implant surgery at a weight range between 350 and 550 g. Animals had free access to food and water during a 7 d recovery period after the surgery, then they were put on a mild food restriction diet (to 90% of free-feeding weight) throughout the experiment period. All procedures were conducted in compliance with the Animals (Scientific Procedures) Act 1986 of the United Kingdom and the European Directive 2010/63/EU on the protection of animals used for scientific purposes. Biological replicates are defined as replications across animals, whereas technical replicates are within-animal manipulations.

### Implant surgery

Before surgery each animal was subcutaneously injected with an analgesic (carpofen 0.5 mg/kg in 2 ml isotonic sterile saline) before being anesthetized and then stereotaxically implanted with tetrodes in either left or right RSC (target coordinates, AP −5.0–5.6 mm, ML ±0.6–1 mm, DV 0.5–0.7 mm, relative to Bregma). The laminar layers of recording were not specifically identified. For half of the animals, the microdrive (Axona) was positioned at an angle of ∼10° towards the midline to sample both dysgranular and granular subregions. Animals received meloxicam 1 mg/kg s.c. for postoperative analgesia for 3 d.

### Experimental apparatus

**Figure 1.**
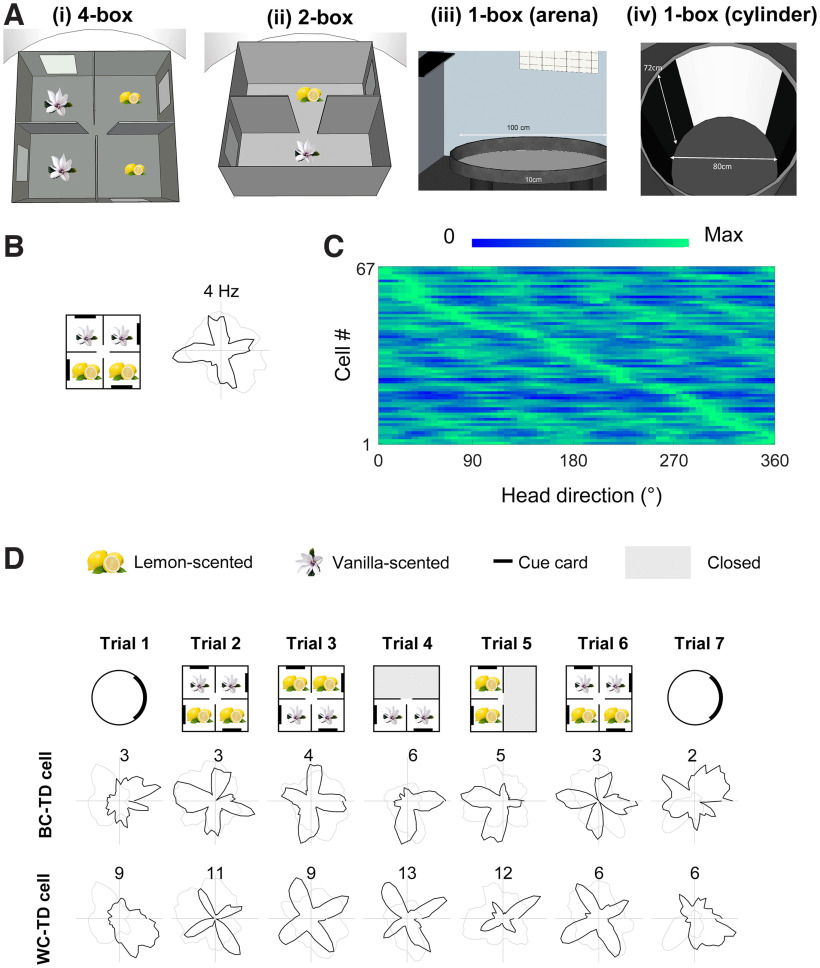
Multidirectional tuning of retrosplenial neurons. ***A***, The four environments used in the study. Curtains surrounded all environments except the open field (***iii***, 1-box arena). ***B***, Example recording from a single trial in the 4-box, showing four-peaked firing on the polar plot of firing rate versus head direction. ***C***, Firing of the population of cells passing the selection criteria for TD firing. Each row is a cell, and color is scaled to that row's maximum. Note the bands of increased firing rate recurring at 90° intervals. Note also that across the population, all firing directions are approximately equally represented. ***D***, Directional firing patterns of two TD cells, a BC-TD cell and a WC-TD cell. These were recorded simultaneously from different tetrodes. Plots show cell activity in standard seven-trial recording sessions; numbers show maximum firing rate in Hz. Note that when restricted to two adjacent compartments (trials 4 and 5) the preferred firing directions are at 90°, regardless of odors.

Recording enclosures of varying symmetry were used in the study (Fig. 1*A*), each placed in the center of a curtained-off, cue-controlled region except for the circular arena, which was open to the room. The 4-box (120 × 120 × 60 cm) contained four equal-size square subcompartments interconnected with a central 10 cm wide doorway. Each subcompartment was polarized by two environmental features—the doorway in the innermost corner and a white cue card (20 × 40 cm) mounted on the far-right-facing wall as seen from the doorway. The spatial relationship between the cue card positions relative to the doorway remained the same for all four subcompartments, forming a 90° rotational symmetry. The global symmetry was broken by scenting one pair of compartments with lemon and the other with vanilla odor using bakery concentrates (Oetker), which our previous experiment found was enough to stably orient the head direction cells (Jacob et al., 2017) so that the directional system would know which way around the entire space was oriented.

For the 2-box, we used the same apparatus as in the previous study (Jacob et al., 2017): two abutting 120 × 60 cm rectangular boxes sharing a long wall that had a central doorway connecting the compartments. Each box was adorned with a single white cue card on one of the short walls, one at the North and one at the South end. This arrangement created a visual 180° rotational symmetry of the global space. The global symmetry was broken, as before with lemon scent in one compartment and vanilla in the other. This also provided a hierarchical clustering of the subspaces in case there might be neurons showing a global directional tuning for a similar pair of subspaces (that is, firing in the same direction for the two lemon compartments and a different direction for the two vanilla ones). We did not see this, however.

For the onefold environments, two circular arenas (1-box) were used, located in two different experiment rooms—a standard open-field arena with low walls, allowing view of the room, and a cylinder with a cue card, which allowed investigations of cue control. The low-walled circular arena (diameter 100 cm, height 10 cm) was used with the 2-box only. It was raised 50 cm from the floor and open to the distal room cues. The high-walled cylindrical arena (diameter 80 cm, height 72 cm) was placed in a different cue-controlled room and used with all 4-box experiments and a for few sessions in the 2-box experiment.

The floor and walls of the apparatus were covered with black vinyl sheets that were cleaned after each session. The multicompartment boxes were placed in the same experimental room, with floor-to-ceiling black curtains surrounding the apparatus to minimize distal cue influence.

### Electrophysiological recording procedures

Before the start of the experiment, animals were acclimated to a separate screening room and were monitored daily for single-unit activity in circular environments. Tetrodes were lowered 50–150 μm after every recording session, or if no cell activity was detected. In all trials, animals were motivated to explore and sample all heading directions in the apparatus as much as possible while foraging for randomly scattered rice or Coco Pops (Kellogg's United Kingdom). Between trials the animal was gently removed from the apparatus, placed in a holding box inside the curtain to prevent its knowledge of any room cues or experimenter's manipulation of the apparatus, and then mildly disoriented inside a custom-built rotating box before the next trial. Experiment room change happened between the trials in the 2-box/4-box and 1-box environments (see below), and the animal was carried between the rooms in an opaque box to prevent perception of any distal cues.

Single-unit activity was recorded using a multichannel data acquisition system (Axona), monitored online with thresholds set by the experimenter. The microdrive was attached to a head stage, which was connected to a recording cable leading to a preamplifier and digitizer (48 kHz). Single-unit signals were amplified 5000–8000 times and bandpass filtered between 300 and 7000 Hz; local field potential (LFP) signals were amplified 1000–2000 times and lowpass filtered at 500 Hz. The head direction and position information of the animal were determined by tracking the positions of two LEDs (one brighter than the other) spaced 5 cm apart. Position and heading were monitored by an overhead camera at a sampling rate of 50 Hz. A radio positioned above the apparatus emitted constant white noise to mask directional auditory cues from beyond the curtains. During recordings, the environmental sound level was maintained over 70 dB to minimize any extramaze auditory influence.

One standard recording session consisted of seven trials, the first and last trial in the 1-box and five multicompartmented box trials in between (Table 1). For trial 1 (1-box baseline), the high-walled cylinder was placed in the center of the room and oriented in multiples of 90° in camera-defined coordinates at the beginning of the trial. The aim of pseudo-random rotation in a cue-controlled room was to check that the cell tuning followed the local cue rotation with the apparatus. The 1-box baseline trials lasted for 10 min for the 2-box experiment and 8 min for the 4-box experiment to ensure adequate behavioral sampling.

**Table 1.** Summary of experiments and trials

Trials	4-box experiment	2-box experiment
1-box baseline	8 min	10 min
Baseline (door open)	16 min	10 min
Rotated	16 min	10 min
Door closed, half box 1	8 min	5 min
Door closed, half box 2	8 min	5 min
Final baseline	16 min	10 min
1-box baseline	8 min	10 min

For trial 2 (door open, box baseline) the apparatus was placed in the center of the room and oriented in multiples of 90° in camera-defined coordinates at the beginning of the trial. Animals were placed in a randomly chosen subcompartment at the beginning of the trial. Recording duration (see Table 1) for door-open box trials was 10 min for the 2-box and 16 min for the 4-box. Trial 3 (door open, rotated box) is the same as in trial 2 except that the apparatus was variably rotated by ±90 or 180°.

For trial 4 (door closed, half box 1), the central doorway was closed so that the animal was restricted to only half of the apparatus. The recording duration for door-closed trials was 5 min for the 2-box and 8 min for the 4-box. The two lemon or two vanilla compartments were either adjacent or diagonal to test for the possibility there might be directional neurons that would be consistent across a two-compartment space if these were unified by a common odor (exposing a hierarchical encoding of subspaces).

Trial 5 (door closed, half box 2) was the same as in trial 4, but the animal was recorded in the other half of the apparatus. Trial 6 (door-open, box baseline) was the same as in trial 2; the door was open, and the apparatus was rotated back to its starting orientation. Trial 7 (1-box baseline) was the same as in trial 1; the animal returned to the circular environment.

All recording sessions included in the study consisted of at least the seven trials as described above. In addition, the following procedures were sometimes performed. To test whether the firing pattern was primarily dependent on vision, in some 4-box recording sessions (*n* = 28), two darkness trials were added after the last door-open box baseline trial with the same recording duration. The animal and cue cards were removed from the apparatus, and the lighting sources in the room were switched off, then the animal was disorientated and reintroduced into the apparatus. The 4-box was randomly rotated before the second darkness trial.

For the rotated cylinder trials, in some recording sessions of the 2-box (*n* = 8) and 4-box experiments (*n* = 43), at least two extra cylinder trials were added following the last cylinder baseline trial to investigate whether the (uni)directionality derives from the visual environment. The rat was removed from the environment and mildly disoriented, the cylinder and cue card were randomly rotated from the baseline orientation, and then the rat was reintroduced into the apparatus.

### Electrophysiological data analyses

#### Cell identification

Single-unit activity was analyzed off-line using an automated spike-sorting algorithm (Klustakwik version 3.0; Harris et al., 2000), packaged in cluster-cutting software with a graphical user interface (TINT, Axona). Single-unit inclusion was manually checked using spike auto- and cross-correlograms. Cell cluster quality was assessed over a recording session consisting of multiple trials recorded from different environments. Before cluster cutting, spike data collected from all trials within a session were compiled to one overall cluster space so that the cluster center of mass remained constant across trials. Several cluster quality metrics were compared between unidirectional and multidirectional cells to check that cluster isolation difference could not explain the findings. We did this by calculating three metrics, the isolation distance, L ratio (both measures make use of the Mahalanobis distance; Schmitzer-Torbert et al., 2005), and refractory period contamination rate (Fee et al., 1996). The refractory period contamination rate was calculated as the number of spikes in the refractory period (2 ms duration) in proportion to the total number of spikes within a trial. None of these measures differed between directional cells and the recording population as a whole.

#### Directionality analyses

The directionality analyses were applied to every single trial of the recording session for individual animals, with no trials ever combined. All directional cells included had a peak firing rate exceeding 1 Hz.

##### HD polar plot

For each trial, all spikes and head direction samples were binned into 60 bins of 6° width, and the number of spikes emitted in each bin divided by the total dwell time for that direction. A boxcar kernel (width 5) was applied to smooth the raw tuning curve. The peak firing rate was defined as the maximal firing rate found in the smoothed tuning curve, and the angular bin of this maximum value was the preferred firing direction of the cell. For multidirectional cells the preferred firing direction was taken as the angle of the biggest peak. We tested the preferred firing directions from all directional cells for nonuniformity with a Rayleigh test (MATLAB function circ_rtest; Berens, 2009).

*Multidirectional scores.* The multidirectional scores reflected the level of spatial periodicity in the tuning curves and were calculated using a similar circular autocorrelation procedure as described previously (Jacob et al., 2017). The tuning curve of each cell was rotated against itself in steps of 6°, and the Pearson's correlation coefficient was recalculated at each step. Periodicity in the tuning curve would result in a sinusoidal modulation of the circular autocorrelogram, and the number of peaks in this autocorrelogram would reflect the order of symmetry in the tuning curve. The BD score tested the strength of twofold modulation and was defined as the difference between the mean correlation coefficient at the angle of the expected peak (±180°) and the average of the expected troughs (±90°). The TD score tested the strength of fourfold modulation and was calculated similarly, but the expected peaks were at ±90 and ±180° and the expected troughs at ±45 and ±135°. Computation of the scores for the shuffled controls (see below) was performed in the same manner.

*Directional specificity.* Directional specificity was assessed using mean resultant vector length (Rayleigh vector length; Giocomo et al., 2014). However, multifold directionality violates the unimodal assumption of the Rayleigh distribution; we thus performed the directionality analysis by angle-doubling, as used previously (Jacob et al., 2017), and angle-quadrupling procedures in which the heading direction of each spike (in radians) was doubled or quadrupled and expressed modulo 2π. In this way, we converted multifold symmetric data into a unimodal distribution (Landler et al., 2018). Rayleigh vector length was then computed from the converted data. For the 1-box, Rayleigh vector length (without angle conversion) was computed for each cell and compared with the 95th shuffle threshold in two 1-box baseline trials. To characterize the narrow unidirectional tuning pattern in the 1-box, the Rayleigh vector lengths of the cell should exceed that of its shuffle in three consecutive 1-box trials.

*Shuffle control procedure.* A shuffling procedure was used to obtain control distributions and chance-level estimation in directionality and egocentric tuning analyses. In the HD analyses, for each permutation trial, the entire spike train of each cell was circularly shifted in either direction by random intervals of at least 20 s (directional analysis) or 30 s (egocentric analysis; the same parameters as used before (Alexander et al., 2020)) relative to the positional/directional data. One hundred permutations were performed for each trial of a given cell in the shuffling procedure. For each permutation, a shuffle HD tuning curve was constructed, and Rayleigh vector length, TD/BD scores, autocorrelograms, cross-correlograms, egocentric firing rate map, and mean resultant length were determined on an individual basis. Thresholds were identified from the overall distribution of the measurements of the shuffled data (e.g., 95th percentile). At a population level, the thresholds of Rayleigh vector length and multidirectional scores were the mean or the median 95th percentile threshold of all recorded cells in three door-open box trials. This is to obtain a robust threshold cutoff; the shuffle procedures were done 478 × 100 × 3 times in the 2-box experiment and 660 × 100 × 3 times in the 4-box experiment. Note that the mean Rayleigh vector length was higher, and the median of multidirectional scores was higher, so more stringent thresholds were taken.

##### Selection criteria

A classic head direction cell was defined as meeting the following criteria in both box-baseline trials: peak firing rate at least 1 Hz and Rayleigh vector length >0.26, the same threshold as used previously (Jacob et al., 2017). For an overall classification of multidirectional cells, a BD-pattern cell was defined as meeting the following criteria: (1) peak firing rate ≥ 1 Hz, (2) BD score exceeding 95th percentile criterion of shuffle, and (3) angle-doubled Rayleigh vector length exceeding either 95% shuffle or 0.18, a value derived from the population threshold (see below). Similarly, a TD-pattern cell was defined as meeting the following criteria: (1) peak firing rate of the cell ≥ 1 Hz, (2) TD score exceeding the 95% shuffle, and (3), angle-quadrupled Rayleigh vector length exceeding either 95% shuffle or a population threshold at 0.15. All criteria above were applied to both baseline trials in the 2-box and 4-box. The same criteria were also used in analyzing cell activity in 4-box darkness trials.

For within-compartment classification, we further analyzed firing of each cell in single compartments in the first baseline trials. In addition to meeting the overall criteria above, a between-compartment (BC) BD cell (BC-BD) was defined as meeting the following additional criteria: (1) Rayleigh vector length (without angle doubling) in each single compartment exceeding 95% shuffle per cell or the mean Rayleigh vector length of single compartments exceeding 0.18 (population threshold) and (2) a BD score in each single compartments not exceeding the shuffle control per cell. A within-compartment (WC) BD cell (WC-BD) needed to meet the following additional criteria: (1) mean angle-doubled Rayleigh vector length of single compartments exceeding 0.18, and (2) a BD score in each single compartment exceeding 0.26 (population threshold).

Similarly, for between-compartment tetradirecitional (BC-TD) cells, the additional selection criteria were the following: (1) Having a Rayleigh vector length (without angle quadrupling) in single compartments exceeding 0.15 (population threshold) in at least three single compartments and (2) having a TD score of single compartments not exceeding the shuffle control. For WC-TD cells the criterion was the TD score and angled-quadrupled Rayleigh vector length of single compartments exceeding 0.15 in at least three single compartments.

In the 1-boxes (circular baselines), cells that were considered broad-unidirectional had areas under the correlation curve exceeding the 95th percentile of its shuffle. Cells with their Rayleigh vector lengths exceeding the 95% shuffle in all three successive cylinder trials were included for comparison analysis with HD cells.

##### Symmetry analyses

For all recorded cells, we autocorrelated their tuning curves in the first baseline trials in the 2-box, 4-box, and their associated 1-box trials. From the autocorrelation, we calculated the area under the curve values for each cell. In addition, to examine the directional pattern (onefold, twofold, and fourfold) across environments and their subcompartments, we calculated the cross-correlation (Pearson's correlation coefficient) between two tuning curves in each angular bin in steps of 6°. For the 2-box, the tuning curve pairs were from lemon and vanilla subcompartments of the baseline trials. For the 4-box, the tuning curve pairs were from any adjacent two subcompartments (i.e., four pairs, vanilla 1 vs. lemon 1; lemon 1 vs. lemon 2; lemon 2 vs. vanilla 2; and vanilla 2 vs. vanilla 1), which were pooled, and the final cross-correlation was averaged over these four comparisons. For 1-boxes, the tuning curve pairs were from two baseline trials. Periodicity would appear as a sinusoidal modulation of the circular cross-correlogram in a range from −180 to 180°, with the number of peaks reflecting the order of symmetry in the tuning curve. Computation for the shuffle control was performed in the same manner. Statistical tests were performed to compare the data and the shuffle.

#### Egocentric spatial tuning analyses

The egocentric analyses were applied to the subset of directionally tuned cells in our experiments. We used the same set of analyses as described previously (Hinman et al., 2019; Alexander et al., 2020). For each cell recorded in an environment, the allocentric position data points of the animal were used to obtain the following: movement direction (instantaneous derivative of successive position samples in the traveling path), the distance from a point to all environment boundaries visible by direct line of sight, and the angle from a point to all visible boundaries (in 3° increments). The spiking activity over the trajectories of the animal was color coded by the its movement directions.

To construct the two-dimensional egocentric boundary rate maps, angular bins were referenced to the current movement direction so that 0° was to the front of the animal, 90° to its right, 180° to its back, and −90° to its left. For every spike, its distance to the walls was computed within a range from zero to half of the length of the most distant possible boundary. The number of distance bins was 20, and the distance bin size and the maximum possible distance varied adaptively for each type of environment because of different environment dimensions (e.g., 40 cm for the circular arena, 50 cm for the square box, 60 cm for the 2-box, and 30 cm for the 4-box). For a given 2D bin, the number of spikes within that spatial bin was divided by the dwell time. Rate maps were smoothed using a 2D Gaussian kernel (width 5).

For the egocentric boundary tuning, we applied the same methods and criteria used previously. The mean resultant (MR) was calculated as follows:

$$MR=\frac{\left(\sum_{\theta =1}^{n}\sum_{D=1}^{m}F_{\theta ,D}\cdot e^{i\text{*}\theta }\right)}{n\cdot m},$$

in which θ is the egocentric bearing to boundaries, *D* is the distance, *F*_θ,*D*_ is the firing rate in a given egocentric spatial bin, *n* is the number of orientation bins, and *m* is the number of distance bins. Then mean resultant length (MRL) was calculated as the absolute value of MR. The mean resultant angle (MRA), the preferred egocentric boundary orientation of a cell, was calculated as follows:

$$\mathrm{MRA}=\text{arctan}2(\frac{\text{imag}\left(\text{MR}\right)}{\text{real}\left(\text{MR}\right)}).$$

The preferred egocentric boundary distance was calculated based on the MR angle by fitting a Weibull distribution to the firing rate along the MRA. The distance bin with the maximal firing rate was taken as the preferred boundary distance. For a cell to be classed as having significant egocentric boundary coding, the MRL would exceed the 99th percentile of the shuffle, preferred distance shift <50% in the first half versus second half of a trial, and MRA would shift <45° in the first and second half of a trial. We applied the above criteria to examine recorded directional cells and calculated the proportion that passed all three criteria in different environments.

#### Basic electrophysiological features

The analyses were performed on the first baseline trial of the multicompartmented boxes for TD-pattern, BD-pattern, and HD cells.

##### Waveform

The waveform width (peak-to-trough latency) and the amplitude were extracted for each cell. We collated the waveform features for all directional cells (*n* = 217) and fitted a two-component gaussian mixture model (GMM) classifier (MATLAB functions fitgmdist, clustering) that assigns each data point to the mixture component corresponding to the highest posterior probability.

##### Burst index

The burstiness of neurons was measured by the spike-burst index, which was defined as the proportion of spikes (of all spikes) that occurred within 6 ms of the interspike interval histograms (Harris et al., 2001; see below).

##### Interspike interval

For each cell, the interspike interval (ISI) of a spike train was analyzed within a time window of 50 ms. An Epanechnikov kernel (bandwidth 6) was fitted to the time interval distribution to get a smoothed density estimate. We calculated the latency between the points where the spiking probability crossed a threshold of half of the maximum, and then compared the population data between cell groups.

##### LFP phase and spiking activity

The LFP analysis methods were established and described in a previous study (Grieves et al., 2020). To obtain a theta phase angle for each spike, the LFP signals were first bandpass filtered in the 6–12 Hz range (fourth-order Butterworth, MATLAB functions butter and filtfilt) before a Hilbert transform was applied to obtain the instantaneous phase angle (MATLAB function hilbert). For each cell, the instantaneous theta phase of every spike was calculated by linear interpolation of the instantaneous theta phase signal (MATLAB function Phase). The phase angles of the cell were binned between −π and π in 0.1 radian bins. The preferred theta phase of the cell was defined as the circular mean of these angles, and the strength of the modulation (phase locking) was defined as the mean resultant vector length of these angles (MATLAB functions circ_mean and circ_r, respectively; Berens, 2009). For each cell, the number of spikes was normalized to its maximum in the baseline trial. At the population level, the average number of spikes per angular bin was calculated, and all cell preferred phases were collated.

### Statistical analysis

We applied the circular statistic toolbox (Berens, 2009), and parametric tests, and *post hoc* nonparametric tests were applied to compare population means and medians. In one-way ANOVA, the Games–Howell *post hoc* procedure was used if the data violated the homogeneity of variance assumption. The statistical tests were performed in MATLAB (R2018, MathWorks) and IBM SPSS 25 packages. Where appropriate, the effect sizes for each test have been stated clearly. All statistical tests are two tailed (*p* value threshold at 0.05) unless stated otherwise.

### Histology

At the end of the experiment, animals were anesthetized with isoflurane and injected with an overdose of sodium pentobarbital for euthanasia. Half of the animals underwent an electrolytic lesion by passing a small 15∼20 µA current through one to two electrical channels for 6 ∼ 12 s before perfusion. After a transcardiac perfusion, the brain was extracted and fixed in cold paraformaldehyde (4%). The brains were sliced using a freezing cryostat (Leica Biosystems) under −21°C, and 35–40 μm coronal sections through caudal extent of the RSC were taken, stored in wells containing PBS, and wet mounted on the slides. The slices best representing the electrode tracks were then imaged using an Olympus microscope (Olympus KeyMed). The deepest point of the electrode track was identified by referencing to the Paxinos and Watson (2006) rat brain atlas. The history of tetrode movements was used together with these points to calculate the tetrode depth for each recording session. The records were used to compare the localization of the recorded cells in dysgranular versus granular subregions in RSC.

### Data availability

Summarized datasets are available on Figshare at https://doi.org/10.6084/m9.figshare.21647423.

## Results

RSC neurons were recorded from 18 rats (Table 2) freely foraging in environments having onefold symmetry (1-box), twofold symmetry (2-box), or fourfold symmetry (4-box). Every cell was recorded in one of the two 1-boxes and in either the 2-box or the 4-box (above, Materials and Methods). We first present the 4-box data, then the 2-box, and then finally the 1-box results, which flanked the multifold box trials (Chronologically, the 2-box experiment was conducted before the 4-box experiment.). To assess possible carryover effects from experience in the 2-box, for the 4-box experiment we used four naive rats in addition to four rats that previously had nine sessions in the 2-box (total rats, *n* = 8; cells, *n* = 743; Table 2, Fig. 1). We finish by comparing the properties of these multidirectionally tuned cells with classic HD cells.

**Table 2.** Data summary of animals and directional cells

Experiment	Rat	All cells		HD cells		MD cells		Recording site
		*n*	% total	*n*	% total	*n*	% total	
4-box	973	9	1.2	1	1.3	0	0.0	gRSC (r)
	974	76	10.2	12	16.0	6	9.0	dRSC, gRSC (l)
	975	68	9.2	2	2.7	1	1.5	dRSC, gRSC (r)
	976	172	23.1	24	32.0	8	11.9	dRSC, gRSC (l)
	978	35	4.7	3	4.0	2	3.0	dRSC, gRSC (r)
	006	178	24.0	19	25.3	39	58.2	dRSC, gRSC (l)
	015	124	16.7	10	13.3	1	1.5	dRSC, gRSC (l)
	027	81	10.9	4	5.3	10	14.9	dRSC, gRSC (l)
**Total *n***	**8**	**743**		**75**		**67**		
2-box	863	13	2.2	0	0.0	1	2.1	dRSC (l)
	872	6	1.0	4	14.8	1	2.1	gRSC (r)
	887	36	6.3	2	7.4	4	8.3	dRSC, gRSC (l)
	889	38	6.6	6	22.2	3	6.3	dRSC, gRSC (r)
	890	85	14.8	4	14.8	6	12.5	dRSC, gRSC (l)
	891	35	6.1	0	0.0	4	8.3	dRSC (r)
	892	135	23.5	5	18.5	6	12.5	dRSC, gRSC (l)
	910	53	9.2	6	22.2	3	6.3	dRSC, gRSC (r)
	911	101	17.6	0	0.0	4	8.3	dRSC, gRSC (r)
	912	18	3.1	0	0.0	7	14.6	dRSC (l)
	973	6	1.0	0	0.0	1	2.1	dRSC (r)
	974	14	2.4	0	0.0	4	8.3	dRSC (l)
	975	22	3.8	0	0.0	3	6.3	dRSC (r)
	976	13	2.3	0	0.0	1	2.1	dRSC (l)
**Total *N***	**14**	**575**		**27**		**48**		

l, left; r, right.

### A new tetradirectional firing pattern in a fourfold symmetric environment

We first looked at how RSC directional cells might behave in the 4-box. Previous modeling work suggested that the retrosplenial bidirectional pattern might break down in a fourfold symmetric environment (Page and Jeffery, 2018), and so we increased the level of environment symmetry from twofold to fourfold (the 4-box; Fig. 1*Ai*). Contrary to our prediction, in the 4-box, in addition to canonical unidirectional HD cells (*n* = 75/743, 10.1%), we saw many cells with a fourfold-symmetric four-leaf clover firing pattern, with a 90° offset between four directional tuning peaks (Fig. 1*B–D*). The fourfold pattern also occurred in three of the four animals with previous 2-box experience (*n* cells = 15), whereas the twofold symmetric pattern, described below, did not. This fourfold directionality was quantified as a TD score; 67/743 (9%) of cells (mean firing rates, 5.0 Hz) met the criteria for tetradirectionality (see above, Material and Methods).

**Figure 2.**
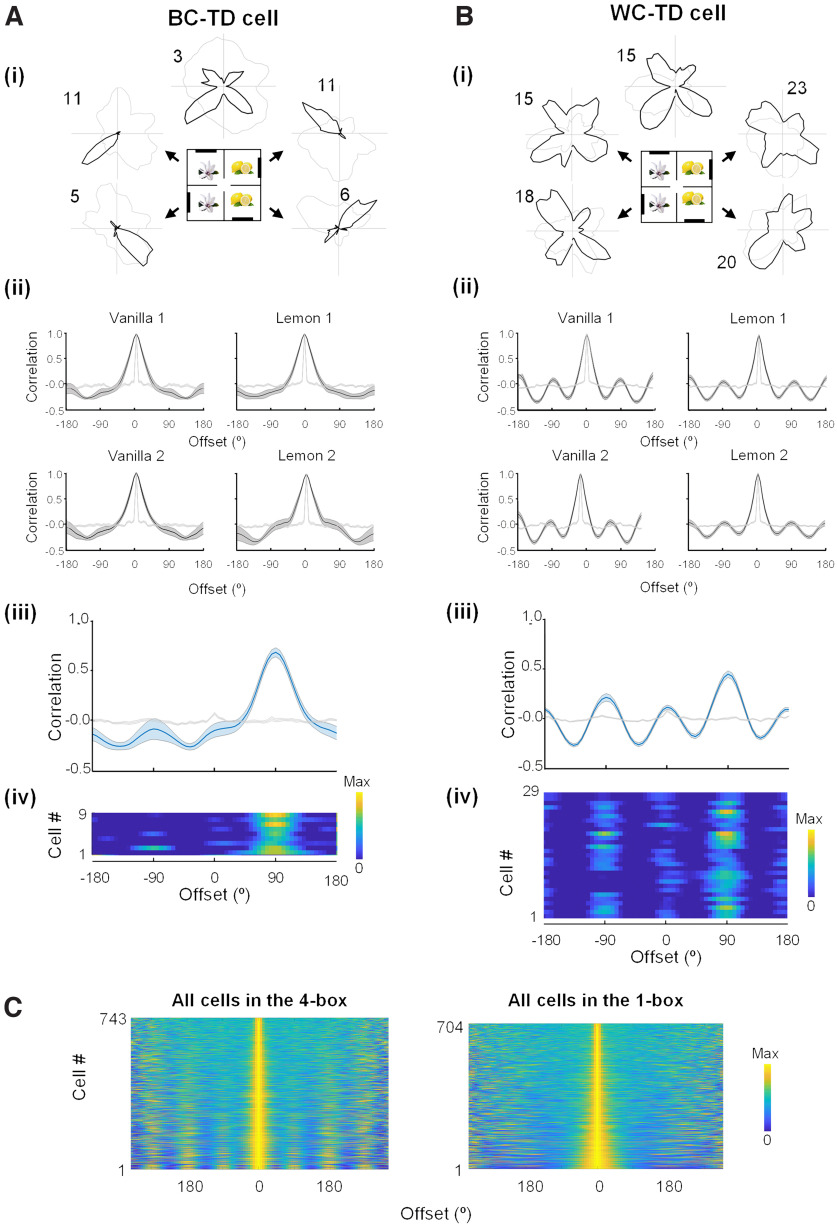
Directional firing symmetries in the 4-box. ***A***, ***B***, A between-compartment tetradirectional cell (***A***), and a WC-TD cell (***B***). For both ***A*** and ***B*** the subplots are as follows. ***i***, Polar plots from individual neurons. Note the occurrence of fourfold (90°) symmetries for the global pattern alone for the BC-TD cell and for both global and local patterns in the WC-TD cell. ***ii***, Rotational autocorrelograms of the tuning curves in each subcompartment (mean ± SEM, dark gray), compared with the shuffled data (light gray). The BC-TD cell has onefold symmetry (single peak) in each compartment, whereas the WC-TD cell has fourfold symmetry (four peaks). ***iii***, Population cross-correlograms of the plots above (each compartment compared with the one clockwise adjacent). For the BC-TD cells, note the single peak at +90°, reflecting the 90° rotation of the tuning curves that followed the 90° rotation of the compartment layouts. For the WC-TD cells there were four peaks, but the one at 90° was larger, reflecting an underlying onefold symmetry. ***iv***, Same data as in ***iii*** shown as heat maps of individual cells, each scaled to its own maximum. ***C***, Heat maps of the autocorrelograms of all cells (not just those passing the selection criteria) recorded from the 4-box experiments, sorted by the area under the curve. Left, The 4-box baseline. Right, The associated 1-box arena. Note the 90° repetition in the 4-box but not the 1-box. Note also that although we used a threshold-determined subset of TD cells in the qualitative analyses, the fourfold-symmetric pattern is evident to some extent in all the cells.

In single subcompartments of the 4-box we identified three subgroups of TD-pattern cells (Fig. 1*D*): unidirectional, multidirectional, and nondirectional. Following our previous convention, we named the unidirectional cells between-compartment tetradirectional (BC-TD) because the tetradirectional pattern only emerged when comparing firing between compartments (Figs. 1*B*, 2*A*). We found 9/67 TD cells (13%) with this pattern. Loss of tetradirectionality within a compartment was shown by TD scores near zero (mean, vanilla 1 = 0.00; vanilla 2 = −0.02; lemon 1 = −0.03; lemon 2 = 0.03; *p* > 0.6 in all cases). Unidirectionality within a compartment was shown by a high Rayleigh vector score (median, vanilla 1 = 0.27; vanilla 2 = 0.22; lemon 1 = 0.25; lemon 2 = 0.32) and single-peaked within-compartment autocorrelations significantly different from a shuffle in every subcompartment (Fig. 2*Aii*; Kolmogorov–Smirnov test, vanilla 1, *D* = 0.67, *p* < 0.001; vanilla 2, *D* = 0.66, *p* < 0.001; lemon 1, *D* = 0.72, *p* < 0.001; lemon 2, *D* = 0.52, *p* < 0.001).

We then cross-correlated the firing patterns in adjacent subcompartments to reveal how orientation followed the compartment layout. Consistent with the rotation of environmental layout from one compartment to the next, the global preferred firing direction in each subcompartment rotated by 90° in successive subcompartments; cross-correlations of tuning curves from adjacent subcompartment pairs peaked at ∼90° (circular *V*-test against 90°, *V* = 7.98, *p* < 0.001) and differed from a shuffle (Fig. 2*Aiii*,*iv*), Kolmogorov–Smirnov test, *D* = 0.68, *p* < 0.001).

We also checked whether there was any bidirectionality in the firing patterns of the kind seen in our previous study, which might reflect an intrinsic propensity of the cells to produce a bidirectional pattern. However, BC-TD cells did not show a bidirectional pattern in single compartments, having BD scores not significantly different from zero (mean, vanilla 1 = 0.07, *t*_(8)_ = 0.51, *p* = 0.62; vanilla 2 = 0.08, *t*_(8)_ = 0.40, *p* = 0.70; lemon 1 = 0.04, *t*_(8)_ = 0.29, *p* = 0.78; lemon 2 = −0.09, *t*_(8)_ = −0.45, *p* = 0.67).

A second subgroup of TD-pattern cells, which we call within-compartment tetradirectional (WC-TD; *n* = 29/67, 43%; Figs. 1*B*, 2*B*) exhibited a multidirectional pattern even in single subcompartments (Fig. 2*Bii*). This was usually fourfold, although occasionally one peak was smaller. The within-compartment fourfold symmetry was revealed by TD scores significantly greater than the shuffle control (mean, vanilla 1 = 0.41, *t*_(28)_= 6.03, *p* < 0.001; vanilla 2 = 0.36, *t*_(28)_ = 4.04, *p* < 0.001; lemon 1 = 0.35, *t*_(28)_ = 5.31, *p* < 0.001; lemon 2 = 0.23, *t*_(28)_ = 1.70, *p* = 0.05). Also, the coefficient of within-compartment autocorrelations was significantly different from shuffle (Kolmogorov–Smirnov test, vanilla 1, *D* = 0.49, *p* < 0.001; vanilla 2, *D* = 0.47, *p* < 0.001; lemon 1, *D* = 0.52, *p* < 0.001; lemon 2, *D* = 0.50, *p* < 0.001).

Despite the fourfold symmetry there was also an asymmetry present; the four tuning curve peaks varied in size, and the cross-correlation analysis showed that the direction of the largest peak usually rotated 90° between adjacent subcompartments, with peaks at ∼90° (Fig. 2*Biii*,*iv*); circular *V*-test against 90°, *V* = 13.69, *p* < 0.001) that significantly differed from a shuffle (Kolmogorov–Smirnov test, *D* = 0.47, *p* < 0.001).

The third group of cells (29/67, 43%) were not directional in individual subcompartments and remained unclassified. We rarely saw cells with only two or three peaks in the whole 4-box (one cell, recorded from one animal that had experience in the 2-box, showed a BD pattern in both 4-box baselines). Moreover, we did not see any cells that were directionally consistent across both lemon or both vanilla compartments but that rotated between lemon and vanilla (i.e., there was no apparent hierarchy of subcompartment encoding).

Note also that although we analyzed a threshold-determined subset of TD cells in the analyses above, the fourfold-symmetric pattern is evident to some extent in all the cells, in the 4-box but not the 1-box (Fig. 2*C*), suggesting a general influence on retrosplenial directional encoding by the environment structure.

**Figure 3.**
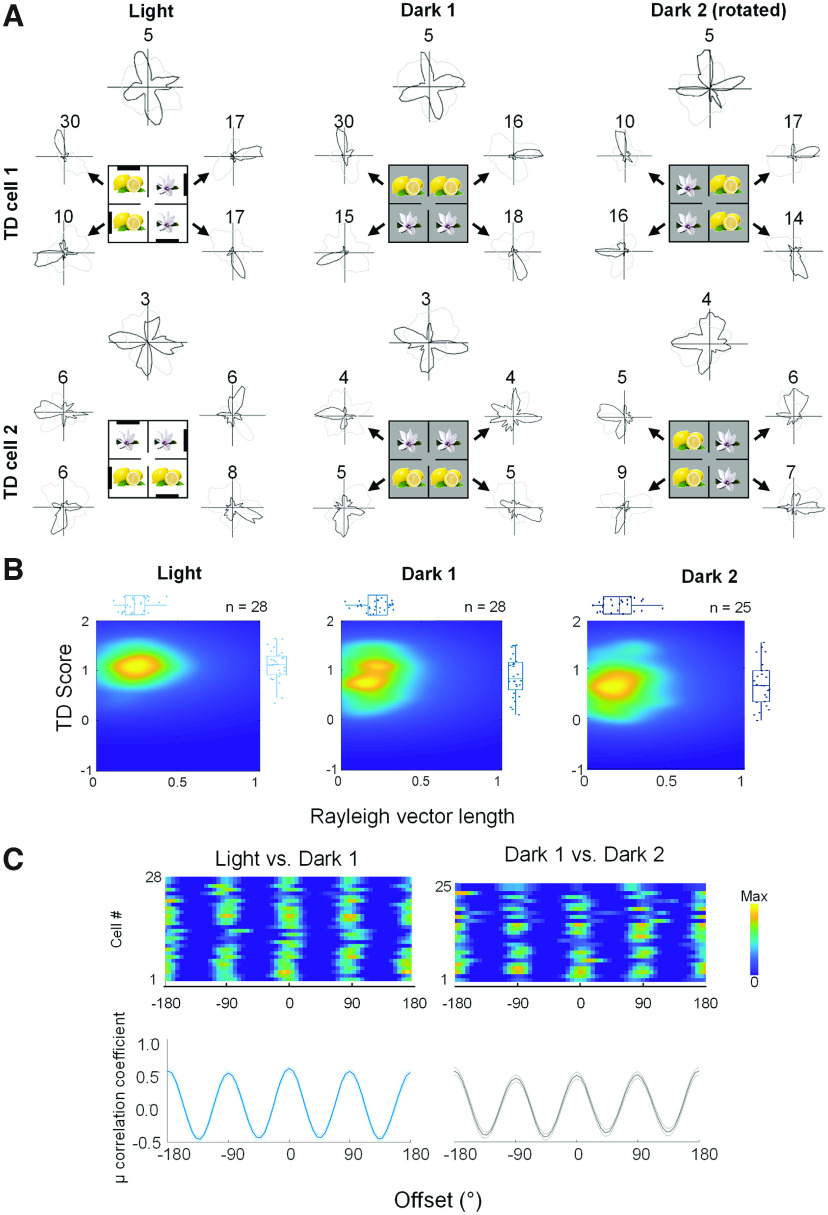
Maintained but decreased TD pattern in darkness. ***A***, Example of two TD-pattern cells that maintained the four-way firing pattern in two darkness trials following the 4-box baseline. Note that the animal was removed from the 4-box, mildly disoriented, and reintroduced to the apparatus before the dark trials started. ***B***, Density and box plots showing distribution of TD scores as a function of Rayleigh vector length in light (first box baseline) and two dark trials, significantly different in TD scores (repeated-measures ANOVA, *F*_(2,78)_ = 5.26, *p* = 0.007) and Rayleigh vector length within the group (*F*_(2,78)_ = 3.36, *p* = 0.04). Between-group comparisons of TD scores, significant for light versus dark 1 (*t* test, *t*_(27)_ = 2.51, *p* = 0.018), but not significant for dark 1 versus dark 2 (*t*_(24)_ = −0.36, *p* = 0.725). Between-group comparisons of Rayleigh vector length: significant for light versus dark 1 (*t*_(27)_ = 4.35, *p* < 0.001) but not significant between two dark trials (*t*_(24)_ = −0.52, *p* = 0.61). ***C***, (Top) Cross correlograms of all TD tuning curves in the light and dark 1 trials (left) and the dark 1 and dark 2 trials (right). (Bottom) The average of these cross-correlations. These did not differ (Kolmogorov–Smirnov test, *D* = 0.165, *p* = 0.142).

We considered whether the symmetric firing pattern might be because of the visual layout of the environments and so made some recordings of TD-pattern cells in the dark in the 4-box (Fig. 3). We found that 75% maintained their four-way pattern in the first dark trial (*n* = 21/28), 64% in the second dark trial (*n* = 16/25), and 44% in both (*n* = 11/25). Together with previous findings of a preserved BD pattern in the dark (Jacob et al., 2017), the results suggest that the multidirectional cells were not solely dependent on immediate visual inputs and that multimodal sensory information could help maintain their directional tuning.

### Bidirectional tuning curves emerged in a twofold-symmetric environment

**Figure 4.**
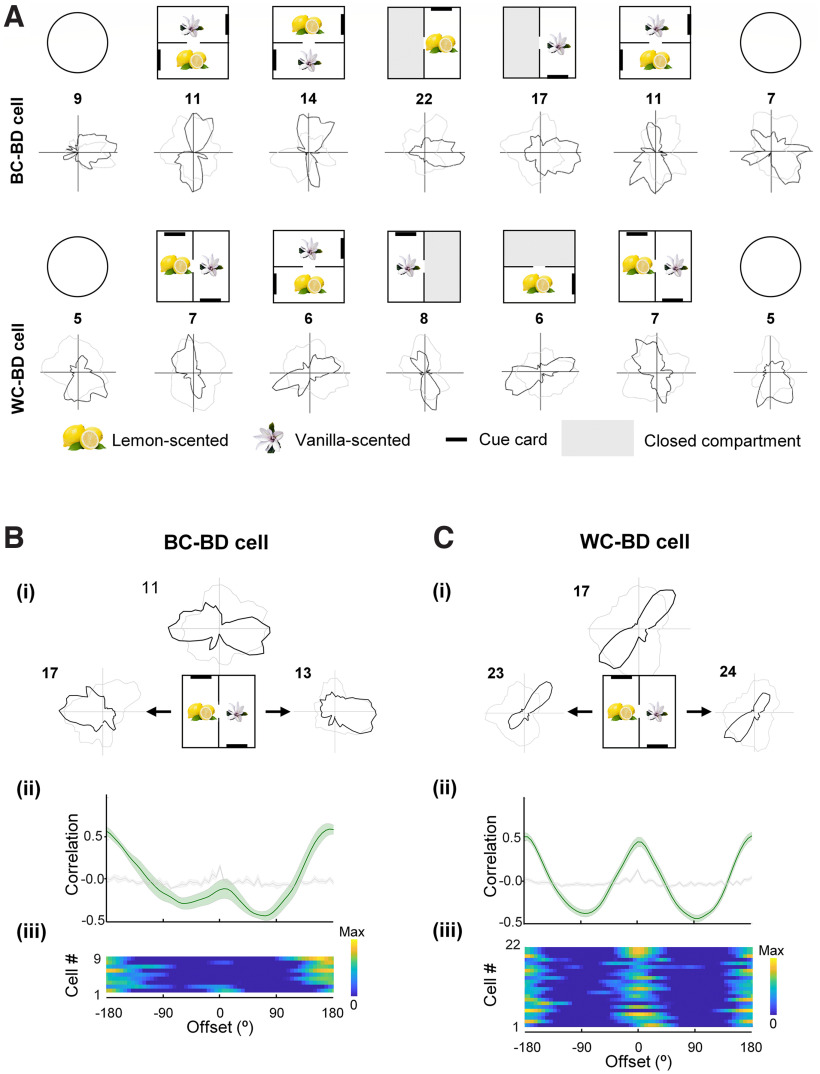
Directional firing symmetries in the 2-box. ***A***, Two example BD-pattern cells recorded from different animals in the 2-box experiment, one BC-BD and one WC-BD. Polar plots show the standard seven-trial recording sessions. Note that in the 1-box (open arena in this case), the firing patterns could be unidirectional or nondirectional. ***B***, ***C***, Symmetry analysis of a BC-BD and WC-BD cell, recorded in single compartments. Subplots are shown as in Figure 2. The BC-BD cell has onefold symmetry in the cross-correlograms (single peak at 180°), whereas the WC-BD cell has twofold symmetry (two peaks, at 90° and 180°).

We compared the results in the 4-box with recordings in the 2-box (Fig. 1*Aii*), which we conducted to replicate our previous results and also to explore whether we could see an emergence of the pattern in the early stages of recording (although as it happened, we could not collect enough data from this phase.). In the 2-box we recorded 575 RSC cells from 14 rats (Table 2). As well as classic HD cells (27/575, 4.7%), and consistent with our previous findings (Jacob et al., 2017), we found directionally tuned cells expressing bidirectional tuning curves,: that is, a twofold-symmetric firing pattern with a 180° offset between two tuning curves (Fig. 4*A*). This was quantified with a bidirectionality score and angle-doubled Rayleigh vector length; 48/575 cells (8.3%; mean firing rates 5.4 Hz) passed the criteria for bidirectionality.

As with the TD cells, these cells displayed three main firing patterns. First, the so-called BC-BD cells (*n* = 9/48, 19%; Fig. 4*B*) showed a different firing pattern within single subcompartments, with a bidirectionality score close to zero (mean, lemon = 0.15; vanilla = −0.12, *p* > 0.3 in all cases) and significant unidirectionality. The latter was shown by a significant Rayleigh vector score (median, lemon = 0.22; vanilla = 0.23), and within-compartment autocorrelations that were single peaked at zero (Fig. 4B*ii*,*iii*) and significantly different from a shuffle (Kolmogorov–Smirnov test, lemon, *D* = 0.65, *p* < 0.001; vanilla, *D* = 0.59, *p* < 0.001). Thus, for these cells only the global pattern was bidirectional (cross-correlations significantly different from shuffle, Kolmogorov–Smirnov test, *D* = 0.58, *p* < 0.001; peaked at 174°, circular *V*-test for concentration at 180°, *V* = 4.76, *p* = 0.012).

Second, the WC-BD cells (*n* = 22/48, 46%; Fig. 4*C*) expressed a bidirectional pattern even within single subcompartments (BD scores above shuffle control; mean, lemon = 0.80; vanilla = 0.68; within-compartment autocorrelations significantly differed from a shuffle; Kolmogorov–Smirnov test, lemon, *D* = 0.52, *p* < 0.001; vanilla, *D* = 0.52, *p* < 0.001). Where the two peaks of the within-compartment tuning curves were asymmetric, the direction of the large/small peaks rotated by ∼180° between the two subcompartments (Fig. 4C*ii*,*iii*); between-compartment correlations were significantly different from shuffle, Kolmogorov–Smirnov test, *D* = 0.51, *p* < 0.001; mean ΔPFD = 188°, circular *V*-test against 180°, *V* = 6.54, *p* = 0.024). A third category of BD-pattern cells (17/48, 35%) fell below the statistical threshold for directionality in the subcompartment analysis and remained unclassified.

### The multidirectional firing pattern is specific to multifold symmetric environments

**Figure 5.**
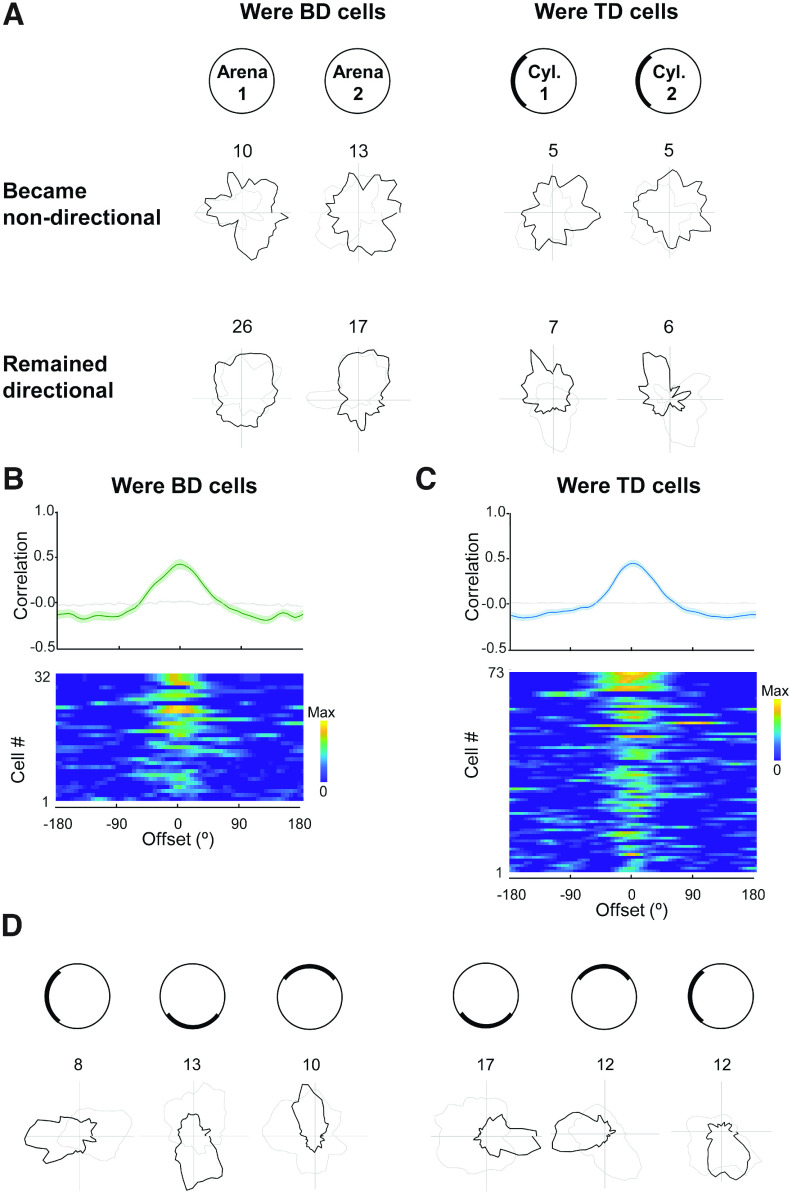
Variable unidirectional firing symmetries in the 1-box. ***A***, Four example cells, two that remained directional and two that lost directionality, each shown in two trials to show the pattern stability. ***B–C***, Population data from former BD cells (***B***) and former TD cells (***C***) ordered top to bottom by Rayleigh vector; format as described in Figure 2, showing the cross-correlation data. The prominent single peak and central stripe indicates preserved unidirectionality in the 1-boxes. ***D***, Two cells, a former BD-pattern cell (left) and former TD-pattern cell (right) that developed singular tuning curves in the 1-box that followed cue rotation, indicating that the directionality of the cells was sensitive (at least in part) from local visual cues.

Henceforth, we refer to BD- and TD-pattern cell collectively as multidirectional (MD). So far, we have shown that MD cells are driven by a multifold environment symmetry, but how would they behave in environments with singlefold symmetry? To answer this question we looked at the onefold-symmetry trials (the 1-boxes, the open arena and cylinder), which flanked the multicompartment trials. We found that although some cells lost directionality (Fig. 5), a significant proportion became unidirectional; this was the case in both the open arena and the cue-controlled cylinder for cells that previously had been either BD or TD cells.

In the open arena, for cells that were BD in the 2-box (*n* = 32; Fig. 5*B*), we found that overall, the cross-correlation between the two baselines had a tall central peak that significantly differed from the shuffle (Kolmogorov–Smirnov test, *D* = 0.63, *p* < 0.001), indicating above-chance directionality at the population level. To quantify this at the level of individual cells, the Rayleigh vector length of each cell's data was compared with that of the same data shuffled in both baseline 1-box trials; 14/32 cells (BD, 44%) had Rayleigh vector lengths higher than the shuffle of the cell in both baseline trials (median, circular baseline 1 = 0.12; circular baseline 2 = 0.13), showing a broad unidirectional pattern; and 56% lost directional specificity. They did not show bidirectional pattern either; only 3/32 cells had BD scores higher than the shuffle.

We undertook a similar analysis for the cue-controlled cylinder 1-box trials in which a single cue card broke the rotational symmetry. Eight cells were formerly BD in the 2-box, and 65 were formerly TD in the 4-box; we pooled these for analyses in the 1-box. At the single-cell level, as before, the Rayleigh vector was compared with the shuffle of the cell in both baseline 1-box trials; 28/73 (38%) cells (26 were TD, 2 were BD) had Rayleigh vector lengths higher than their shuffle in both baseline trials (median, circular baseline 1 = 0.17; circular baseline 2 = 0.18), and 45/73 (62%) cells lost the directional specificity. They did not show a multidirectional pattern in 1-box trials; either only 1/8 had a BD score higher than shuffle, and 3/65 cells had TD scores higher than the shuffle in both trials. As a population, the cross-correlations of the cells between the two 1-box baselines showed a single central peak (Fig. 5*C*) that was significantly different from shuffle (Kolmogorov–Smirnov test: *n* = 73, *D* = 0.67, *p* < 0.001). Confirming the sensitivity to local visual cues, some cells responded to cue rotation (Fig. 5*D*), although this was inconsistent, perhaps because of familiarity of the rats with the room context.

### Multidirectional firing does not arise from egocentric boundary relationships

One possible explanation for our results might be because animals are constrained by environmental geometry to experience nonhomogeneous relationships to the environment boundaries. If the cells are sensitive to these boundaries, this might show up as nonhomogeneous firing rates that cluster around particular directions. For example, because animals in a North–South-aligned rectangle tend to move North–South more often than East–West, if a cell is tuned to a boundary to the left (or right) of the animal, this might cause more firing in the North–South direction than the East–West direction and produce bidirectional tuning. Indeed, Alexander et al. (2020) suggested that this type of egocentric boundary coding, which they found in RSC, might explain our bidirectional cells.

**Figure 6.**
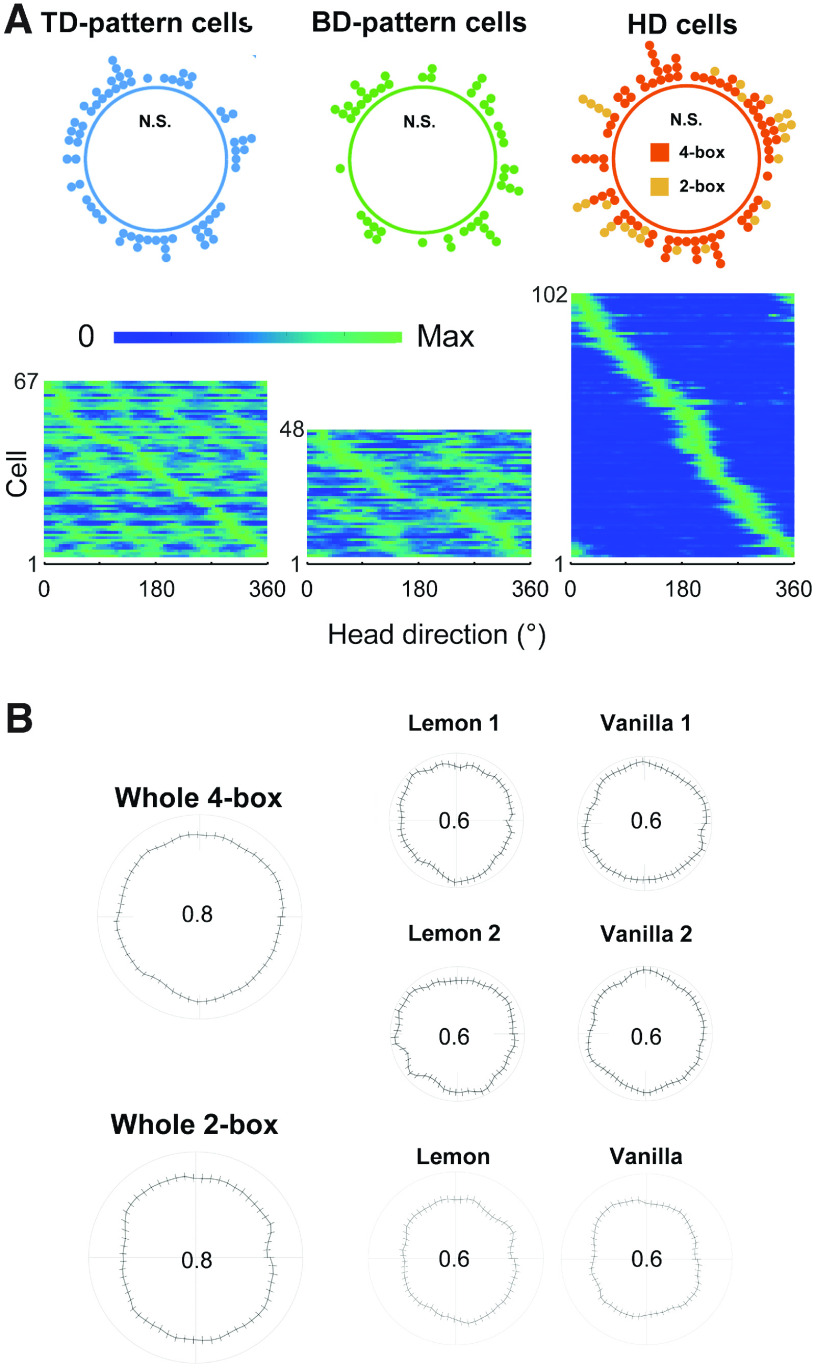
Homogeneous distribution of directional tuning and behavior. ***A***, Allocentric directional tuning of retrosplenial cells was homogeneously distributed in the multifold-symmetric environments. Top, Circular dot plots show that the preferred orientations of the multidirectional cells, like classic HD cells, were distributed homogenously across the whole environment. Bottom, The tuning curves are shown as heat plots. ***B***, Aggregated polar plots of directional dwell time (curve, mean of sessions in which directional cells were recorded: the small ticks are error bars that indicate SEM); in the whole and individual single boxes, showing that behavioral directional biases did not account for directional firing.

To investigate this possibility, we undertook two sets of analyses. First, as a general survey, we looked at the directional distribution of tuning curves for cells in all three environmental symmetries to see whether there were inhomogeneities that mirrored the symmetries in the environment. This might occur because in multisymmetric environments some egocentric boundary vectors are only available in some directions. For example, in a 1-m-square box, vectors with a >1 m distance component would only be available in directions with the symmetry of the diagonals, which is fourfold, so firing in cells tuned at these distances might look tetradirectional. Similarly, in a rectangular box, cells with egocentric tuning distances longer than the short axis might appear to have directional firing preferences with the symmetry of the long axis (twofold) and thus fire bidirectionally. However, this spurious directionality should cluster in the directions of the most common egocentric boundary preference, which for RSC has been reported to be at around 90° to the left and right of the animal (Alexander et al., 2020). In contrast, we found the directional firing distributions to be uniform around the circle (Fig. 6*A*; Rayleigh test, TD, *z* = 0.76, *p* = 0.47; BD, *z* = 0.63, *p* = 0.53; HD, *z* = 0.04, *p* = 0.96). This was also the case for the directional dwell times (amount of time spent facing each direction; Fig. 6*B*). There were no inhomogeneities in direction for each of the environments as a whole or for the subcompartments (4-box, whole apparatus, *z* = 0.02, *p* = 0.981; lemon 1, *z* = 0.001, *p* = 0.996; vanilla 1, *z* = 0.02, *p* = 0.982; lemon 2, *z* = 0.003, *p* = 0.997; vanilla 2, *z* = 0.02, *p* = 0.977; and 2-box, whole apparatus, *z* = 0.02, *p* = 0.982; lemon 1, *z* = 0.06, *p* = 0.941; vanilla 1, *z* = 0.001, *p* = 0.999). Overall, then, it does not seem that inhomogeneous directional sampling likely accounts for the peaked tuning curves.

**Figure 7.**
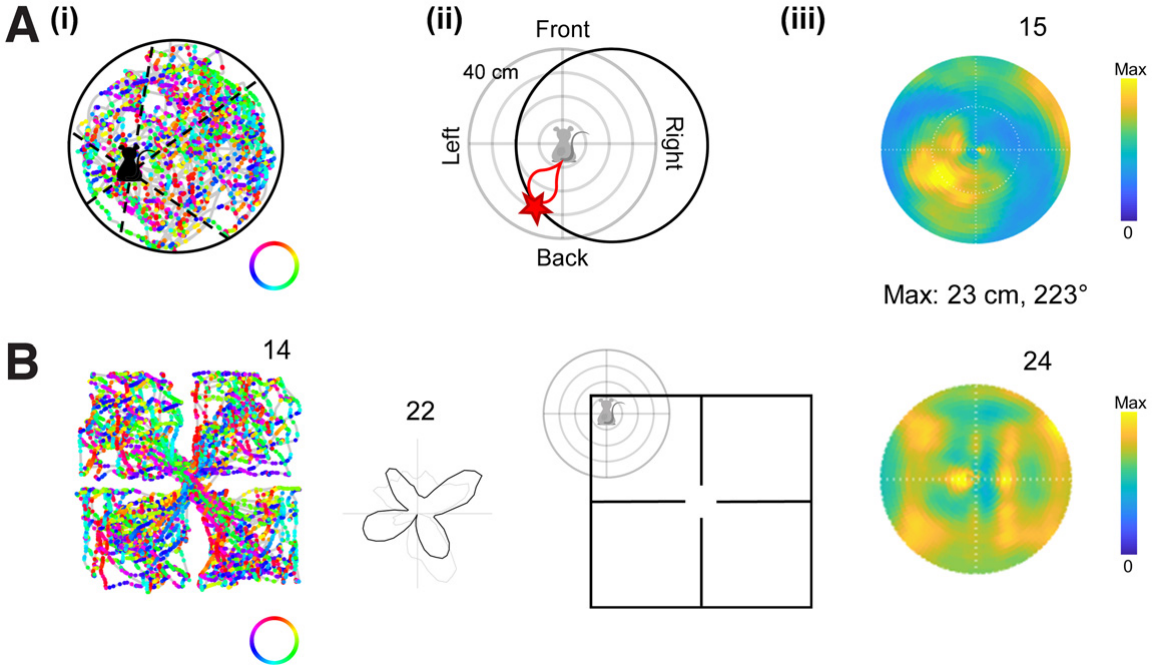
Multidirectional cells were not egocentrically tuned in any environment. ***A***, Schematic of the 1-box analysis illustrating egocentric firing map construction, using one of the only four cells that passed the EB criterion. ***i***, Spike plot of firing as a function of allocentric movement direction. Individual spikes are color coded by the movement direction of the animal. Bottom right, Directional color key shown in the annulus. At each time step the distance is calculated between the position of the rat (black shape) and the boundary, for every direction in 3° bins (6 examples shown by the black dotted lines). ***ii***, Calculation of EB vectors. The egocentric reference frame of the rat is shown in gray, with the animal at the center; the black circle shows, at a given moment, the location of the arena boundary relative to this reference frame. The red teardrop shows a hypothetical EB field of a cell, emanating a given distance and direction from the rat. When the rat moves to a place where the boundary intersects this field (red star), then the cell spikes. ***iii***, Heat plot of the egocentric firing field of the example cell, being the spiking rate as a function of boundary location in the egocentric reference frame. Top right, The number denotes the maximum firing rate, whereas the other text shows the distance and direction, in egocentric coordinates, of the firing peak. This cell showed significant EB tuning, but most did not. ***B***, The EB field of a more typical TD-pattern cell that did not show significant egocentric tuning in the 4-box. Note the absence of obvious fourfold spatial symmetry in the spike plot. This kind of pattern represents the majority of the cell population.

Our second analysis addressed more directly the issue of egocentric boundary (EB) coding, by assessing each cell individually to see whether its firing is related to the location of a boundary at a specific distance and egocentric direction. Using previously published methods based on the direction of movement (Alexander et al., 2020; Fig. 7*A*) we computed EB fields for cells in two baseline trials in the 1-box and multicompartment boxes. The vast majority of directional cells did not display an evident EB firing field or pass the EB criterion (see Fig. 7*A* for an, example of one of the four cells that did); the majority of cells did not show any clear peaks (Fig. 7*B*). However, significantly more by-chance EB firing in a single trial was seen in the MD cells than in the HD cells (chi-square test, TD vs HD, χ^2^(1, *N* = 169) = 15.92, *p* < 0.001; BD vs HD, χ^2^(1, *N* = 150) = 17.62, *p* < 0.001; TD vs BD, χ^2^(1, *N* = 115) = 0.16, *p* = 0.691). This may be a statistical artifact because of the cells' being more responsive to multiple directions.

### Comparisons of MD cells with HD cells

**Figure 8.**
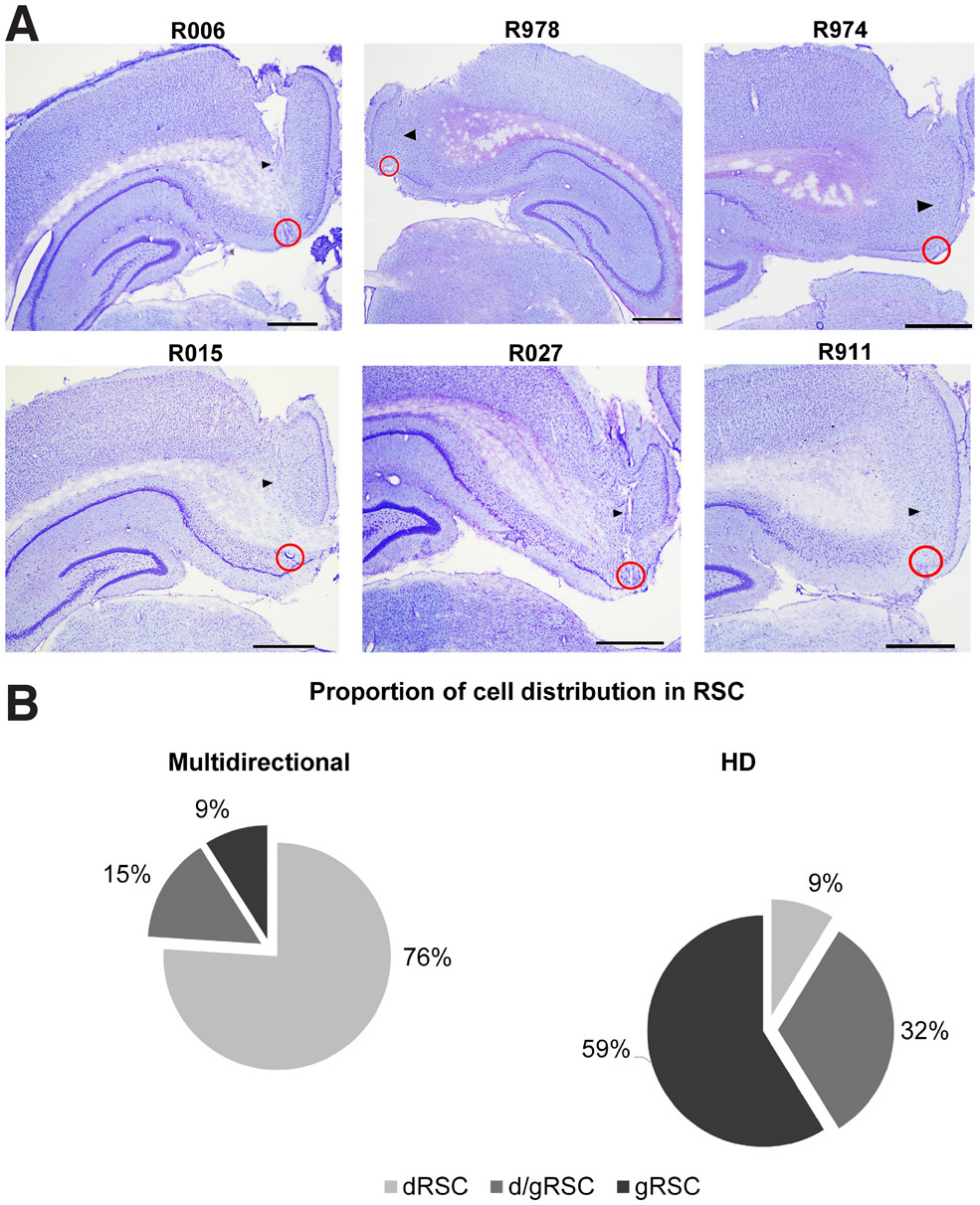
Different distributions of multidirectional and HD cells in RSC subregions. ***A***, Example histologic slices of six animals implanted with bundles of eight tetrodes (R006, R978, R015, R027) or four tetrodes (R974, R911). Animals received a small electrolytic lesion under anesthesia before perfusion, producing visible brain tissue damage at the tip of electrodes (red circles). Scale bar, 1 mm. Black arrows point to the trajectories of the electrode bundles. The median recording coordinates were AP, −5.5; ML, ±0.83 of all animals (*n* = 18). ***B***, Proportion of directional cells in the different subregions of RSC (multidirectional cells, *n* = 115; HD cells, *n* = 102).

Finally, we investigated whether MD cells may be a physically separate neuronal subclass from classic HD cells in RSC by looking at low-level anatomic and physiological properties. First, we looked at anatomic distribution. Similar to previous results (Jacob et al., 2017), a significantly higher proportion of MD cells (76%) than HD cells (9%) was found in the dysgranular RSC (Table 2; Fig. 8; chi-square test, χ^2^ (2, *N* = 217) = 101.89, *p* < 0.001).

**Figure 9.**
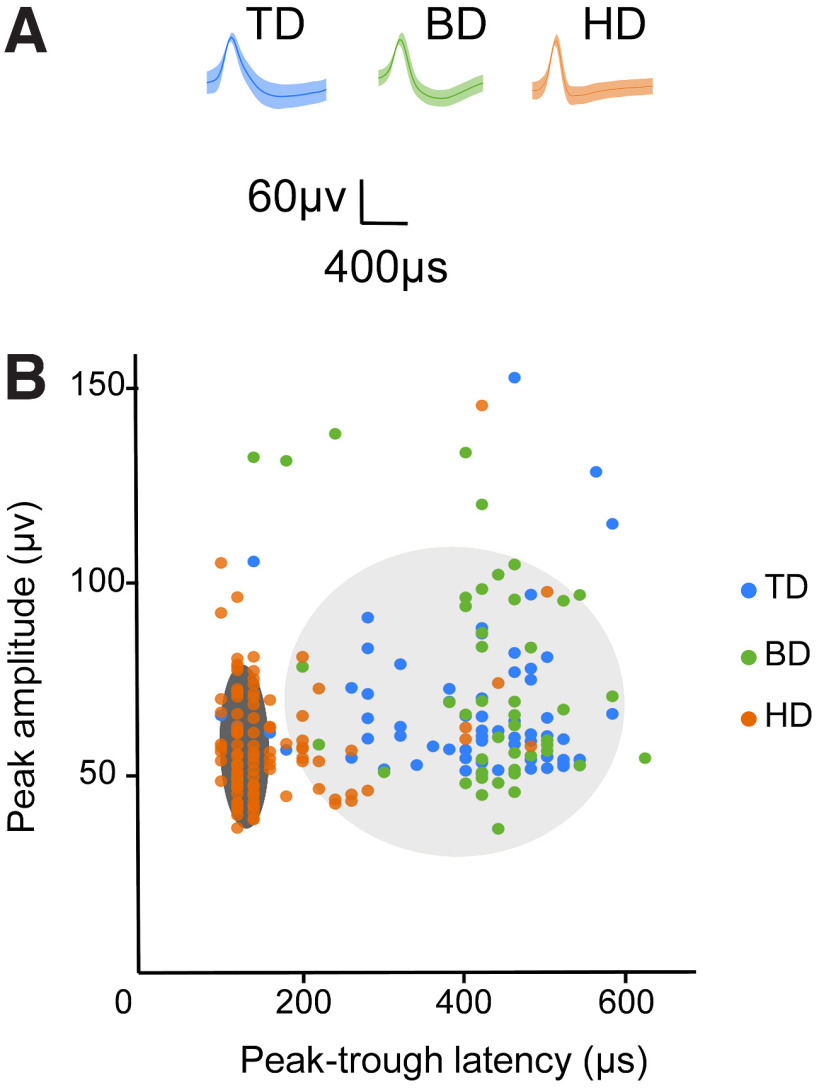
Electrophysiological properties of MD cells are distinguishable from HD cells. ***A***, ***B***, Waveforms of multidirectional cells are taller and broader than those of classic HD cells. ***A***, Example waveforms (mean ± SEM). ***B***, Scatter plots show all directional data (colored dots) fitted with a two-component GMM. Two cluster memberships were plotted as two ellipsoids (with a 99% probability threshold for each confidence region), dark gray for cluster 1 (*n* = 83) and light gray for cluster 2 (*n* = 134), overlaid with recorded cell data. Most HD cells (75.5%) were in cluster 1, and most multidirectional cells (94.8%) were in cluster 2.

Second, we found that the waveforms of MD cells in general were taller and broader (Fig. 9*A*,*B*) with higher peak amplitudes (ANOVA, *F*_(2,101.87)_ = 7.84, *p* = 0.001), longer peak-trough latencies (*F*_(2,100.98)_ = 159.73, *p* < 0.001), and longer latencies to the interspike interval half-peak (*F*_(2,94.87)_ = 33.64, *p* < 0.001). We also did an unbiased clustering of all the waveforms and fitted these with a two-component GMM (Fig. 9*B*). The two fitted clusters were significantly different in their peak-trough latency (*t*_(215)_ = 20.66, *p* < 0.001) and peak amplitude (*t*_(215)_ = 3.98, *p* < 0.001). Of the 102 HD cells, 77 were in the low-latency cluster 1, whereas of the 115 MD cells, only 6 were in cluster 1 (χ^2^(1, *N* = 217) = 113.0185 *p* < 0.00,001). From the waveform features, it remains inconclusive whether RSC HD cells are fast-spiking interneurons, but interestingly, Keshavarzi et al. (2022) reported that ∼20% of their recorded RSC HD cells were putative inhibitory cells.

**Figure 10.**
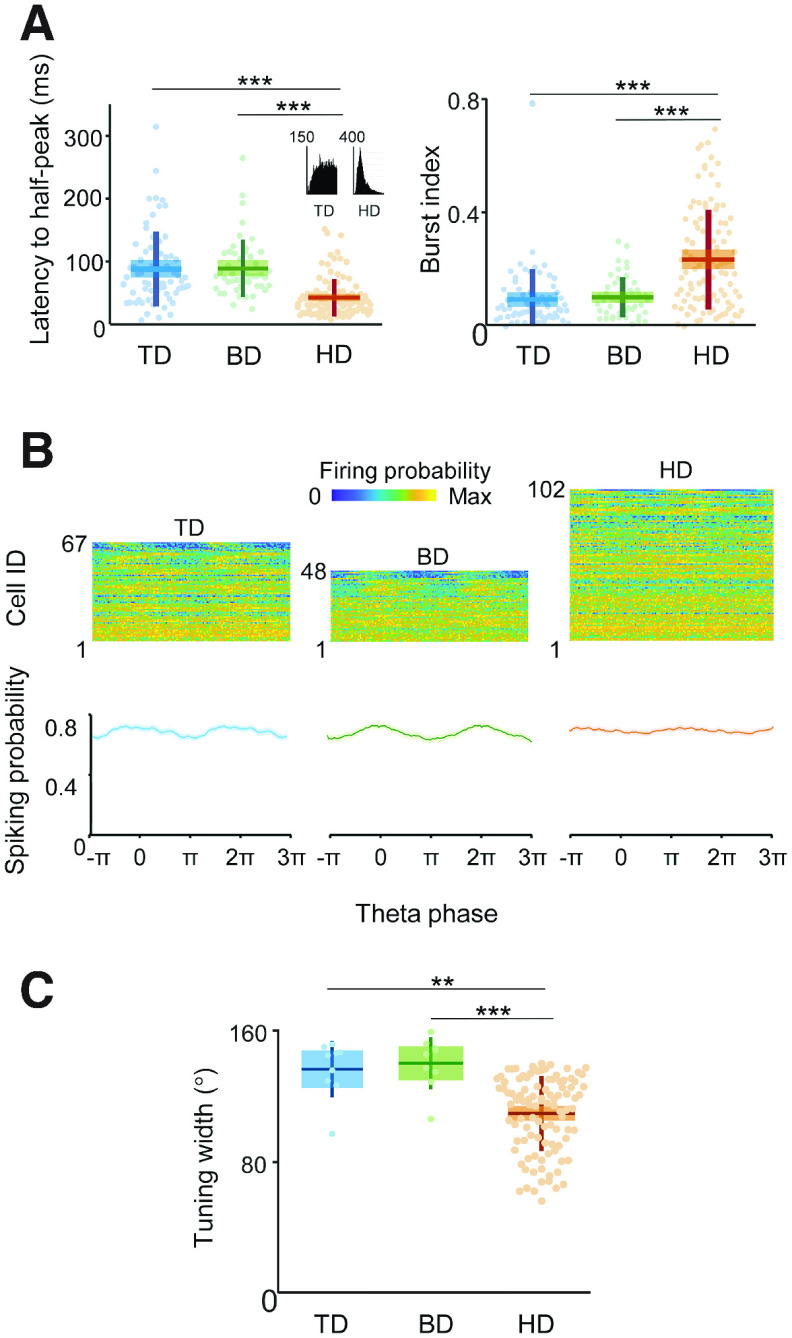
Spiking patterns in time and space differed between MD and HD cells. ***A***, Left, Box plots of latency to interspike interval (ISI) half peak. Inset, An example of the ISI histogram from 0 to 50 ms for a TD and HD cell, showing the much taller, narrower distribution for the HD cell. Right, Box plots of burst index. ***B***, Top, Heat maps show spiking activity as a function of theta phase for the three directional firing patterns. Cell ID was sorted by Rayleigh vector lengths (maximum at top), representing the strength of phase locking. Bottom, Normalized spiking activity (mean ± SEM) over two theta cycles. Theta modulation did not differ by cell class. ***C***, Comparison of tuning width of a subset of multidirectional and HD cell in single-compartment trials of the multifold boxes. *p < 0.05, **p < 0.01, ***p < 0.001.

Third, we looked at the temporal firing characteristics of the cells. HD cells had a higher firing rate, evidenced in interpsike intervals of around half those of the TD and BD cells and were also significantly burstier than MD cells (Fig. 10*A*; Table 3). There was a degree of modulation of some cells by theta phase (Fig. 10*B*), but this did not differ between cell types (Table 3). The results thus indicated an electrophysiological distinction between MD cells and HD cells in spiking patterns but not theta phase relationships.

**Table 3.** Electrophysiological firing properties of directional cells

Neural properties	TD pattern	BD pattern	HD cells	Welch's ANOVA	*Post hoc* comparisons (Games–Howell test)	Effect sizes (Cohen's *d*)
Peak amplitude (µV)	68.51 ± 2.35	74.69 ± 3.94	60.40 ± 1.64	*F*_(2,101.87)_ = 7.84	TD vs BD, *p* = 0.374	TD vs BD, *d* = −0.27
				*p* = 0.001	TD vs HD, *p* = 0.015	TD vs HD, *d* = 0.46
					BD vs HD, *p* = 0.004	BD vs HD, *d* = 0.69
Peak-trough latency (µs)	402.39 ± 13.72	405.83 ± 17.51	162.75 ± 7.99	*F*_(2,100.98)_ = 159.73	TD vs BD, *p* = 0.987	TD vs BD, *d* = −0.03
				*p* < 0.001	TD vs HD, *p* < 0.001	TD vs HD, *d* = 2.54
					BD vs HD, *p* < 0.001	BD vs HD, *d* = 2.54
Burst index	0.10 ± 0.01	0.10 ± 0.01	0.23 ± 0.02	*F*_(2,140.61)_ = 24.81	TD vs BD, *p* = 0.926	TD vs BD, *d* = −0.07
				*p* < 0.001	TD vs HD, *p* < 0.001	TD vs HD, *d* = −0.9
					BD vs HD, *p* < 0.001	BD vs HD, *d* = −0.87
Latency to interspike interval histogram half peak (ms)	88.17 ± 7.28	89.67 ± 6.62	42.12 ± 2.91	*F*_(2,94.87)_ = 33.64	TD vs BD, *p* = 0.987	TD vs BD, *d* = −0.03
				*p* < 0.001	TD vs HD, *p* < 0.001	TD vs HD, *d* = 1.05
					BD vs HD, *p* < 0.001	BD vs HD, *d* = 1.34
Theta modulation strength	0.07 ± 0.01	0.08 ± 0.01	0.05 ± 0.005	*F*_(2,108.37)_ = 2.98	TD vs BD, *p* = 0.523	TD vs BD, *d* = −0.22
				*p* = 0.055	TD vs HD, *p* = 0.252	TD vs HD, *d* = 0.25
					BD vs HD, *p* = 0.07	BD vs HD, *d* = 0.44
Tuning curve width (°)	133.97 ± 5.42	138.66 ± 4.73	109.38 ± 2.29	*F*_(2,14.55)_ = 11.33	TD vs BD, *p* = 0.794	TD vs BD, *d* = −0.31
				*p* < 0.001	TD vs HD, *p* = 0.004	TD vs HD, *d* = 1.08
					BD vs HD, *p* < 0.001	BD vs HD, *d* = 1.30

The assumption of homogeneity of variances was violated, and so Welch's ANOVA was used as an alternative to one-way ANOVA. A Games–Howell test was used for *post hoc* analyses, and Cohen's *d* was used to indicate effect sizes.

Finally, we looked at the directional firing properties, comparing TD and BD cells with HD cells recorded in single compartments. HD cells had narrower tuning curves (Fig. 10*C*). This suggests that although the multidirectional had become unidirectional in the single compartments of the multifold boxes, these cells were not the same as HD cells.

## Discussion

Our data show that a subpopulation of directionally tuned RSC neurons expresses an experience-dependent multidirectional code that reflects the rotational symmetries of the extended environment. In addition to the well-known classic HD cells with globally unipolar, narrow directional tuning, a subset of electrophysiologically distinct directionally tuned cells had firing patterns that reflected the overall environmental symmetry, being unidirectional in a single compartment or bidirectional in a twofold-symmetric environment or tetradirectional in a fourfold-symmetric environment. Furthermore, although some cells expressed these multidirectional patterns globally, being unidirectional in single compartments but multidirectional overall, others expressed them locally. Two questions arise from these findings; what generates these firing symmetries, and what, if any, functional consequences does this have?

### What generates directional firing patterns?

The symmetric firing patterns seem to arise from the symmetries in the environment, but how? The responsiveness to environment layout indicates processing of local sensory cues, and the proximity of RSC to visual cortex suggests that vision might be the dominant sensory modality, providing information about the cue card and the doorway, which together polarized each subcompartment. However, multidirectional firing persists in the dark, which suggests an influence of other modalities, for example, tactile processing of the doorway. Also, with HD cells it is likely that firing in the absence of vision is supported by self-motion cues, which can bridge the gap between sensory resetting events such as contacting the door. The route for this supporting information might be the HD cells themselves, which receive both environmental and self-motion information (Yoder et al., 2011). This leads to a proposed model in which RSC supplies environment information to the HD system, and the latter in return supplies RSC with a computed decision about facing direction that comprises a weighted combination of both information types (Page and Jeffery, 2018; Yan et al., 2021).

Regardless of exact sensory modality, it is interesting to speculate on how the symmetries of the environment can come to influence the firing of the cells. We considered whether multidirectional firing might be a function of local symmetries within the subcompartments, such as the fourfold symmetry of the square boxes. For example, tetradirectional cells that were unidirectional in a single compartment (the BC-TD cells) might have been sensitive to the onefold-symmetric cues in the single compartments—the door and the cue card—thus generating onefold symmetric patterns. By contrast, the cells that remained tetradirectional in single compartments (WC-TD cells) might have been insensitive to these polarizing cues but sensitive to the corners or the walls or both, which had fourfold symmetry. In support of this hypothesis of local environment responsiveness, recent studies have reported bidirectional tuning patterns in the medial entorhinal cortex (Kornienko et al., 2018) and the postrhinal cortex (LaChance et al., 2022).

That said, we actually do not think that the multidirectional firing arose from such local, within-compartment symmetry processing. For one thing, the distribution of preferred firing directions was uniform around the circle and not clustered as the cues were (Fig. 6*A*). However, this is true also for head direction cells, which we know are sensitive to solitary visual cues, so there need not be a direct relationship between the relevant cues and the direction of the tuning curve of a cell. The more compelling argument is that these within-compartment symmetries only appeared after the experience of the global symmetry; they have never been seen in singular rectangular or square subcompartments. For example, in a previous study we found only single peaks in a twofold-symmetric single compartment (Lozano et al., 2017) and Alexander et al. (2020) also did not report multidirectionality in single compartments. We thus do not think that the multidirectional pattern arises as a result of local symmetry/geometry. The only explanation that fits all the observations is that these within-compartment patterns were learned as a result of the global-environment experience. However, we found that some cells that later became multidirectional had expressed onefold directional tuning in the first 1-box baseline. This suggests some preconfigured responsiveness to local environment layout, which is then elaborated, by learning, into tuning curves that therefore become symmetric if the environment is symmetric.

Multidirectional firing was also found not to be because of egocentric boundary vector coding. We suspected this would be the case because we did not see any of the expected spatially symmetric firing (e.g., firing patterns having fourfold spatial symmetry arising from the fourfold symmetry of boundaries of the square compartments. However, to rule this out properly, we did a detailed analysis of firing as a function of egocentric boundary vector relationships. This confirmed that boundary vector coding did not exceed chance levels. The directional symmetries we saw therefore evidently are learned: they arise from experience of the different environment symmetries. However, they frequently persisted in the onefold-symmetric subcompartments afterward (in the within-compartment patterns), suggesting a learning process that was initially informed by the global exploration but then came to affect local activity.

We considered whether the learning process in one environment might have carried over into another. Although we were not able to record individual cells in both the 2-box and 4-box, we did record from four rats in both conditions, so we looked for carryover effects of previous experience of the animals, that is, cells in the 4-box with bidirectional tuning, acquired after experience in the 2-box. We did not see any hint of bidirectional tuning in these rats, and we therefore think that the firing patterns are context specific. This makes sense from an environment-coding perspective as generalization would imply interference between experiences, which would be detrimental to spatial mapping. Context specificity might be enabled by the inputs coming into the retrosplenial area from the subicular region of the hippocampal formation; these inputs preferentially target the granular RSC (Meibach and Siegel, 1977; Lomi et al., 2021).

Within-compartment expression of an across-compartment symmetry could potentially arise from plastic interactions with the HD system (Page and Jeffery, 2018, describe a model of how this could occur.). Canonical HD cells use self-motion information to establish their globally stable directional orientation (Taube and Burton, 1995), whereas the noncanonical multidirectional cells are sensitive to the environment layout (Jacob et al., 2017). A consequence of multidirectionality is that different populations of HD neurons become coactive with MD neurons, depending on the global orientation of a given subcompartment. This coactivation could allow new HD inputs to form by Hebbian association, which then drive within-compartment multidirectionality.

We considered whether there are limits to the symmetries potentially expressible by these neurons. The four tuning peaks we saw in the fourfold-symmetric environment exceeds a proposed theoretical maximum of three peaks (Page and Jeffery, 2018). Our previous modeling predicted that directional specificity should break down with higher symmetries because of the tuning curves possibly overlapping. However, in this model we had to guess at the width of the attractor bump in the (presumptive) ring attractor, and it may be that we guessed too broadly. The fact that we saw fourfold symmetry in the present experiment suggests potential constraints on the parameters of the ring attractor. The question then arises on whether encoding of higher symmetries (such as sixfold or eightfold) is possible. At the recording level it would eventually become difficult to observe such symmetries because of the widths of tuning curves, which would mean that for practical purposes they would merge. This leads us to wonder whether the apparently nondirectional tuning curves seen in many neurons are actually cells that have acquired so many symmetries (e.g., if their preferred landmarks recur at many different directions in their preferred environments) that the pattern is lost to an observer.

A potential role for intracellular, or perhaps juxtacellular, recording would be to explore our finding that MD cells and HD cells seem to be physiologically distinct populations. Because the HD cells had the narrowest waveforms and high firing rates, they fit the profile of the narrow fast-spiking interneurons found in both dRSC and gRSC by Brennan et al. (2020) in a patch-clamping study of mouse RSC. However HD cells have typically been thought to be excitatory (Skaggs et al., 1995), and excitatory HD cells have been directly observed in presubiculum layer 3 (Preston-Ferrer et al., 2016). Nevertheless the possibility remains that in RSC the HD cell population is inhibitory, which is not inconsistent with some models of ring attractor architecture (Song and Wang, 2005). This would mean that the MD cells would likely correspond to the regular spiking or low rheobase cells also seen by Brennan et al. (2020), although neither population was restricted to dRSC. Further work in awake animals with precise neuronal recording will be needed to pin down these identities.

### What are the functional consequences of this symmetry-influenced firing pattern?

These multidirectional cells form part of a growing collection of neurons in the juxtahippocampal cortex that have directional properties that deviate from those of classic head direction cells, being more strongly influenced by environmental landmarks. For example, LaChance et al. (2022) reported cells in postrhinal cortex that showed directional tuning in response to a single landmark and bidirectional tuning in response to two landmarks. These cells are different from the bidirectional cells in our RSC neurons because we do not see these bidirectional patterns within single compartments in naive animals. It may be therefore that the postrhinal signal is earlier in the landmark processing pathway and more closely related to vision, whereas in RSC the signal shows more influence of HD input which can, because of the attractor dynamics, suppress a second peak (unless the cells have acquired additional attachments to HD cells at different directions in the manner described above). Another bidirectional firing pattern was reported by Kornienko et al. (2018) in medial entorhinal cortex and parasubiculum; again, these patterns were seen in single-compartmented environments and were responsive to visual cue manipulations. Collectively these neurons may form part of a scene-processing network that functions to link views of the environment with allocentric directions (Byrne et al., 2007; Page and Jeffery, 2018; Epstein and Baker, 2019; Yan et al., 2021).

A novel property exhibited by the multidirectional neurons in our experiments is their ability to capture symmetries in the environment via learning. It is interesting to speculate on what the consequences of these firing symmetries might be, functionally speaking. On the one hand, it may be a by-product of having explored a space with multiple similar-looking but differently oriented compartments, forcing an unusual plasticity between the visual layout and the HD cells that confused the system. If this is the case then we might expect that multicompartment environments with repeating elements might produce a confused and disordered cognitive map. It has previously been shown that repeating compartments that have the same alignment are confused by both the place cell system and by animals as a whole (Spiers et al., 2015; Grieves et al., 2016). Our present data raise the possibility that repeating compartments at different orientations might also have potential for confusion, although the preservation of a global HD signal also allows for the possibility of disambiguation. Preliminary work in rats indeed suggests that these compartments can be disambiguated.

This raises the alternative possibility, that this symmetry encoding may have a positive functional consequence for cognitive mapping. The within-compartment MD cells reflect the re-expression, locally, of a global symmetry experienced when exploring multiple compartments and understanding their relationships. This re-expression may make up part of a cognitive mapping system that functions to encode information beyond immediate perceptual reach.

How could this work? One possibility is that it consists of a compression process to more efficiently store complex structural information. Our experimental layout had a highly unusually repetitive structure that allowed us to discern the environmental motifs to which cells were sensitive. In a more naturalistic environment, assuming that different cells are sensitive to different structural elements and that any repeating elements (such as corners, doors etc.) would be irregularly distributed in directional space, the resultant population activity might look rather heterogeneous to an experimenter and yet contain a detailed plan of the global layout that could be use by other cognitive mapping neurons. How such a code could be read out, however, is not obvious.

Overall, our results show that the subset of RSC directional neurons that become multidirectional in spaces with higher-order rotational symmetries form a different subpopulation than classic head direction cells and may play a role in mediating between the perception of local spatial features and construction of a cognitive map.
